# A meta-analysis of the therapeutic effect of probiotic intervention in obese or overweight adolescents

**DOI:** 10.3389/fendo.2024.1335810

**Published:** 2024-01-30

**Authors:** Yuanqing Duan, Lanping Wang, Yan Ma, Lei Ning, Xinhuan Zhang

**Affiliations:** ^1^ Department of Endocrinology, The Second Affiliated Hospital of Shandong First Medical University, Taian, Shandong, China; ^2^ Department of Operating Room, The Second Affiliated Hospital of Shandong First Medical University, Taian, Shandong, China; ^3^ Department of Case Room, The Second Affiliated Hospital of Shandong First Medical University, Taian, Shandong, China

**Keywords:** probiotic, obesity, overweight, adolescent, meta-analysis

## Abstract

**Background & aims:**

Existing evidence on the possible effects of probiotics on obese or overweight adolescents has not been fully established. Therefore, the aim of this study was to explore the effects of probiotic supplementation on anthropometric indices, inflammatory markers and metabolic indices in obese or overweight adolescents.

**Methods:**

The literature up to March 2023 related to probiotic intervention in obese or overweight adolescents was searched and screened from multiple databases, including the CNKI(China national knowledge infrastructure), CBM(Chinese biomedical literature database), PubMed, EmBase, and Cochrane library databases. All randomized controlled trials using probiotic supplements in obese or overweight adolescents were included in this systematic review and meta-analysis.

**Results:**

A total of 8 studies that met the inclusion criteria were included in this study. There were 201 cases in the experimental group (probiotic treatment) and 190 cases in the control group. Compared to the control group, probiotic intervention in adolescents resulted in a decrease in body mass index, fasting blood glucose and C-reactive protein with WMD(Weighted mean difference) and 95% CI of -2.53 (-4.8 to -0.26) kg/m^2^, -0.80 (-1.13 to -0.47) mol/L and -0.24 (-0.43 to -0.05) mg/L, respectively. No significant changes were found in weight, waist circumference, waist-to-hip ratio, insulin, Homeostatic Model Assessment of insulin resistance, interleukin 6, tumor necrosis factor alpha and so on; however, an unfavorable elevated effect in total cholesterol, triglycerides, and low-density lipoproteins was detected with WMD and 95% CI of 0.06 (0.02 to 0.09) mmol/L, 0.18 (0.14 to 0.21) mmol/L, and 0.19 (0.18 to 0.20) mmol/L, respectively.

**Conclusion:**

According to our results, probiotic supplementation was beneficial in managing metabolic indicators such as fasting blood glucose, body mass index and inflammation-related C-reactive protein in overweight or obese adolescents. Further large scale studies are warranted to confirm present findings and to identify the effects and mechanisms to provide more precise evidence for clinical intervention.

**Systematic review registration:**

doi: 10.37766/inplasy2024.1.0081, identifier INPLASY202410081.

## 1 Introduction

The prevalence of overweight and obesity in adolescents has risen dramatically in recent decades and has become one of the most important public health problems (1, 2). Adolescence is a unique transition period accompanied by significant physiological and psychological changes. Obesity-related comorbidities may negatively impact adolescent growth and developmental trajectories, while the rising prevalence of obesity in adolescents is associated with an increase in adult-onset diseases (e.g., type 2 diabetes mellitus, hypertension, nonalcoholic fatty liver disease, obstructive sleep apnea, cancer, and dyslipidemia) (3, 4). As the importance of gut flora is further demonstrated, an increasing number of studies are focusing on the correlation between gut microbiota and obesity (5).

The gut microbiota is the most complex ecosystem in nature, possessing large bacterial populations in the gut (6). A significant correlation between obesity and the specific composition of the gut microbiota has been demonstrated (5).

The gut microbiota is not stationary, and short-term changes can occur through diet and lifestyle changes (7). Studies have shown that the microbial imbalances associated with obesity can be re-established with probiotics and a balanced dietary regimen (8). Currently, the number of studies on the effects of probiotics on obesity is small and mainly based on animal models. The effects of probiotics on human host metabolism, particularly on obesity, have been controversial because of the paucity of research data, inconsistent findings and lack of long-term follow-up results, as well as a serious lack of consistency in terms of strain type, sample size, dosage parameters, treatment duration, and mode of administration, which has hampered comparative analyses between studies and made their safety and efficacy for the treatment of obesity an ongoing controversy. Several studies have shown that probiotics not only lead to weight loss and improved obesity but also have a positive effect on metabolic parameters such as blood glucose, systemic inflammation and energy intake (9). Despite the existing data suggesting that probiotics have significant therapeutic potential in obesity, there are still many hurdles to overcome before probiotic therapy can be recognized in the medical practice of adolescent obesity, and there is controversy and uncertainty regarding the use of probiotics in adolescent obese patients (10).

New medical studies are being published continuously and clinicians are faced with increasingly large amounts of new information, to the point where it has become nearly impossible for clinicians to read and evaluate all available data in a medical field. In addition, the study results are not consistently reproducible and the results of individual studies are often insufficient to provide confident answers (11). Consequently, clinical decision making is particularly difficult when the published data are conflicting or the sample size is too small to be reliable (11). Evidence-based medicine is the best available evidence in the medical literature (12). Moreover, the best evidence in evidence-based medicine is from meta-analyses, which provide a less biased, more precise estimate on a clinical issue (13). Meta-analyses is a statistical technique for combining the results from different studies on the same topic (14), and is becoming popular for resolving discrepancies in clinical research. As such, a meta-analysis is an objective, quantitative synthesis of research findings (14) that increases the statistical strength and precision for estimating effects by combining the results of previous studies and, thus, overcoming the problem of small sample sizes and inadequate statistical strength (15). A meta-analysis can explore the sources of heterogeneity, and identify subgroups associated with the factor of interest, potentially providing new insights for future studies (15).

The purpose of this study is to further clarify whether probiotics can be recommended for the treatment of overweight or obesity in adolescents and to provide more scientific and evidence-based medical evidence for clinical interventions for the disease.

## 2 Methods

### 2.1 Literature retrieval strategy

The PubMed, Embase, Cochrane Library, CNKI, Wanfang and CBM databases were searched to obtain relevant randomized controlled trials published up to March 2023. We used the keywords and subjects both in Chinese and English as follows: “adolescent or adolescent or minors or teen or teenager”; “obesity or overweight”; “probiotics or probiotic or synbiotics”.

### 2.2 Selection criteria

The inclusion criteria were as follows: ①The study participants were obese or overweight adolescents; ②The intervention was probiotic treatment; ③Posttreatment means and standard deviations were directly available in the literature, or posttreatment-related means and standard deviations could be obtained by formula calculation; ④The type of study was a randomized controlled trial.

The exclusion criteria were as follows: ①The study participants were not adolescents; ②Noncontrolled trials, animal experiments or *in vitro* experiments; ③Literature with incomplete data or where the original data statistics could not be extracted; ④Conference papers, abstracts, reviews or meta-analyses; ⑤Secondary obesity with a clear etiology; ⑥For duplicate reports or multiple publications targeting the same subject, the literature with the most recent publications and the most complete data was selected.

### 2.3 Quality assessment

All included studies were randomized controlled studies; therefore, the Cochrane Risk Assessment Tool was used to assess the quality of the included studies. The scale consists of six dimensions (1): random sequence generation (2); allocation concealment (3); blinding (4); incomplete outcome data (5); selective reporting; and (6) other bias. If all of the above criteria are “adequate”, there is a low likelihood of bias; if one of the criteria is “unclear”, there is a moderate likelihood of bias; if one of the criteria is “inadequate” or “not used”, there is a high likelihood of bias.

### 2.4 Data extraction and management

Relevant data were extracted by 2 independent investigators through joint discussion to determine final inclusion, with a third investigator assisting in resolution if necessary. The relevant data were extracted, including author, year and country of publication, target population, number and mean age of participants, intervention duration and type. Additionally, the mean and standard deviation (SD) of the effect indicators of concern at the end of the intervention were extracted.

For each parameter, we used the mean and SD of the postintervention values for the probiotics and control groups, as the study population was randomly grouped for inclusion in the literature after reviewing the references, and as the inclusion literature was clearly stated, we considered that there was no difference between the initial data for the experimental and control groups.

### 2.5 Statistical analysis

R 4.1.1 software is used to calculate the merger effect. Heterogeneity between studies was assessed using the *Q* test and the *I^2^* test. If *P*>0.05 and *I^2^* <50%, a fixed effects model was used; if *P*<0.05 and *I^2^* ≥ 50%, indicating greater heterogeneity, sources of heterogeneity were then explored through sensitivity and subgroup analyses. Finally, publication bias was assessed by Begg’s and Egger’s tests. The WMD or SMD(Standardized mean difference) and 95% CI of the individual studies combined were recorded, and forest plots were used to characterize the results of the individual studies.

## 3 Result

### 3.1 Literature screening process and results

**Figure 1:**
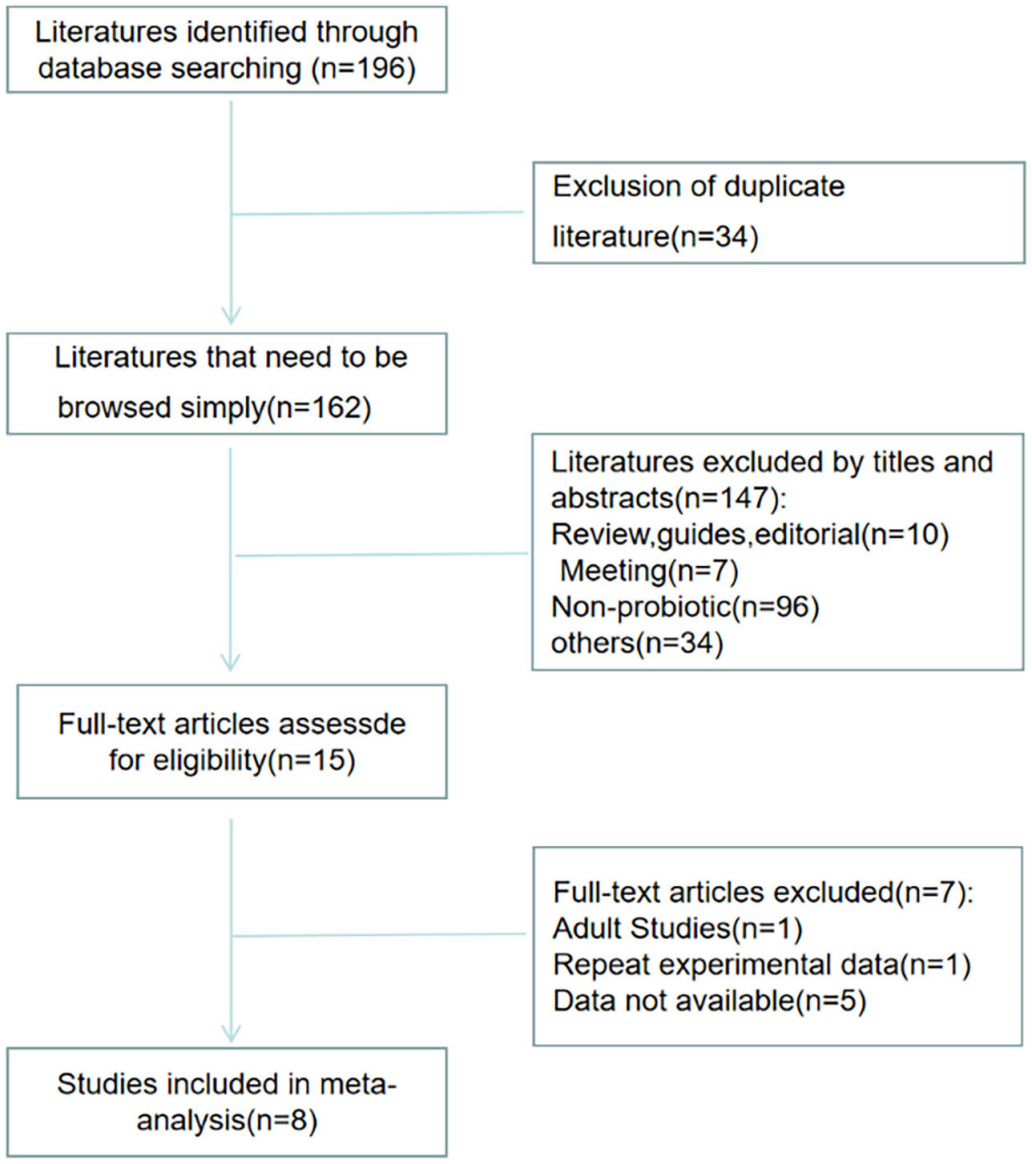
Literature screening flow chart.

**Figure 1** shows the selection process of the studies. There were 196 studies relevant to the search strategy, of which 34 were duplicates. A further 147 of these articles were excluded by browsing the titles and abstracts. After evaluating the full texts of the remaining studies, another 7 articles were excluded. Finally, 8 articles containing 201 cases and 190 controls were included in this meta-analysis (16–23).

### 3.2 Quality evaluation results

**Figure 2:**
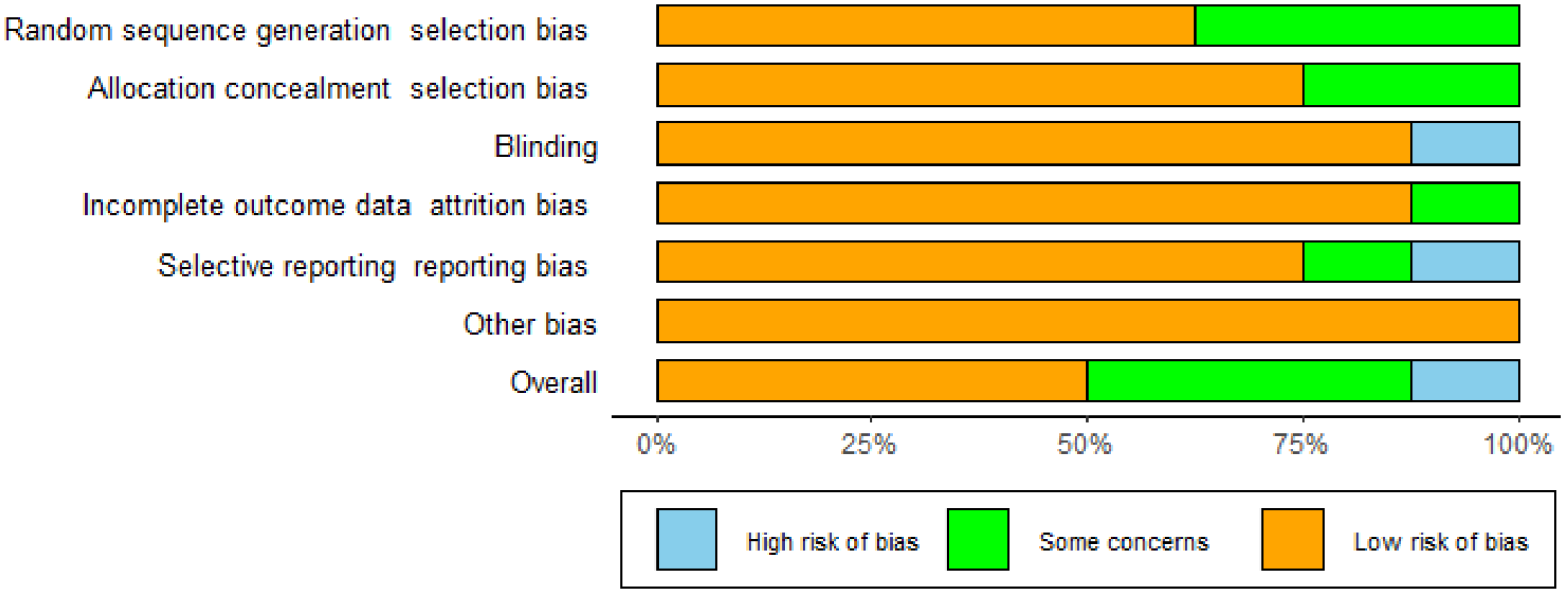
Risk of bias for inclusion in studies.

The studies we included were all randomized controlled studies, so the risk of bias evaluation was performed using the Cochrane Risk Assessment Tool, which was used to assess the quality of eight publications. The randomized allocation of participants was mentioned in all included trials. However, only five trials described randomized sequence generation methods (16, 17, 19, 21, 23), and six trials reported allocation concealment (16, 17, 19, 21–23). With the exception of Ipar’s study (20), which used an open-label design, the remaining seven studies had a low risk of blinding bias among participants, researchers, and outcome assessors. Based on incomplete outcome data and selective outcome reporting, most studies showed a low/unspecified risk of bias, and no other sources of bias were identified. Based on the above assessment, among the eight papers we included in the study, there were four low-bias papers, three medium-bias papers, and one high-bias paper. The risk of bias assessment is detailed in **Table 1** and **Figure 2**.

**Table 1:** Risk of bias assessment for inclusion in randomized controlled clinical trials.

Study	Random sequence	Allocation concealment	Blinding	Incomplete data	Selective reporting	Other bias	outcome
Gobel (2012) (23)	①	①	①	①	①	①	L
Safavi (2013) (22)	③	①	①	①	①	①	M
Kelishadi (2013) (21)	①	①	①	①	①	①	L
Ipar (2015) (20)	③	③	②	①	②	①	H
Famouri (2017) (19)	①	①	①	③	①	①	M
Jones (2018) (18)	③	③	①	①	③	①	M
Verma (2021) (17)	①	①	①	①	①	①	L
Yildirim (2022) (16)	①	①	①	①	①	①	L

① adequate; ② inadequate; ③ unclear L-low-bias M-medium-bias H-high-bias

### 3.3 Characteristics of included studies

Details of the included studies are shown in **Tables 2** and **3**. We extracted the following data from the literature: author, year, country, age of study subjects, sample size, intervention, duration, mean and standard deviation of the investigated outcomes, where some articles did not provide standard deviation, but only sample size and standard error, which we obtained by Equation 1.

**Table 2:** Basic characteristics of the included literature.

author, (year)	Country	sample volume	age	experimental group	control group	duration	Investigated outcomes
Gobel (2012) (23)	Denmark	50(27/23)	12-15	probiotics	placebo	12 w	Weight,WC,WHR,Body fat%,CRP,IL-6,TNF-α,FC,FBG,RI,HOMA-IR,TG, TC, LDL,HDL,
Safavi (2013) (22)	Iranian	56(29/27)	6-18	synbiotics	placebo	8 w	FBG,TG, TC, LDL,HDL
Kelishadi (2013) (21)	Iranian	56(29/27)	6-18	synbiotics	placebo	8 w	Weight,WC,WHR, CRP,IL-6,TNF-α
Ipar (2015) (20)	Turkish	77(42/35)	4-17	synbiotics + Lifestyle modification	Lifestyle modification	30 d	Weight, BMI, WC, CRP, TG, TC, LDL, HDL
Famouri (2017) (19)	Iranian	64(32/32)	10-18	probiotics	placebo	12 w	WC,TG, LDL,HDL
Jones (2018) (18)	USA	19(8/11)	12-18	probiotics	placebo	16 w	BMI, WC,Body fat%
Verma (2021) (17)	USA	8(4/4)	13-19	probiotics	placebo	12 w	Weight,BMI, CRP,FC,FBG,RI,HOMA-IR
Yildirim (2022) (16)	Hong Kong	61(30/31)	8-17	synbiotics	placebo	12 w	Weight,BMI,WC,WHR,FBG,RI,HOMA-IR,TG,TC,LDL,HDL

Synbiotic are prebiotics + oligosaccharides + vitamins; BMI, body mass index; WC, waist circumference; WHR, waist-to-hip ratio; CRP, C-reactive protein; IL-6, interleukin 6; TNF-α, tumor necrosis factor alpha; FC, fecal calprotectin; FBG, fasting blood glucose; RI, insulin; HOMA-IR, Homeostatic Model Assessment of insulin resistance; HDL, high-density lipoprotein; LDL, low-density, lipoprotein; TC, total cholesterol; TG, triacylglyceri.

**Table 3:** Some details of the included literature.

author, year	Probiotic Types and Dosages	control group	duration
Gobel, 2012 (23)	Lactobacillus salivarius, 10^10^ CFU	placebo	12 w
Safavi, 2013 (22)	Lactobacillus Casei, Lactobacillus Rhamnosus, Streptococcus Thermophilus, Bifidobacterium Breve, Lactobacillus Acidophilus, Bifidobacterium Longum and Lactobacillus Bulgaricus, 2.0 ×10^8^CFU	placebo	8 w
Kelishadi, 2013 (21)	Bifidobacterium,Lactobacillus,Streptococcus thermophilus, and prebiotics, 2.0 ×10^8^CFU	placebo	8 w
Ipar, 2015 (20)	Lactobacillus acidophilus (4.3×10^8^CFU), Lactobacillus rhamnosus(4.3×10^8^CFU),Bifidobacterium bifidum (4.3×10^8^CFU), Bifidobacterium longum (4.3×10^8^CFU), and Enterococcus faecium (8.2×10^8^CFU), and Lifestyle modification	Lifestyle modification	30 d
Famouri, 2017 (19)	Lactobacillus acidophilus ATCC B3208,3×109CFU Bifidobacterium lactis DSMZ 32269, 6×10^9^ CFU; Bifidobacterium bifidum A TCC SD6576, 2×10^9^ CFU; Lactobacillus rhamnosus DSMZ 21690, 2×10^9^ CFU	placebo	12 w
Jones, 2018 (18)	VSL#3	placebo	16 w
Verma, 2021 (17)	Vivomixx®, is a mixture of Lactobacilli and Bifidobacteria strains	placebo	12 w
Yildirim, 2022 (16)	Lactobacillus acidophilus(4.3x10^8^ CFU), Lacticaseibacillus rhamnosus (4.3x 10^8^ CFU), Bifidobacterium bifidum (4.3x10^8^CFU), B. longum (4.3x10^8^ CFU), Enterococcus faecium (8.2 x 10^8^ CFU)	placebo	12 w


(Equation 1) $$\text{SD}=\text{SE}\times \sqrt{N}$$

### 3.4 The effect of probiotics in obese or overweight adolescents

#### 3.4.1 The effect of probiotics on anthropometric indices (weight, BMI, WC, WHR, body fat%)

**Figure 3:**
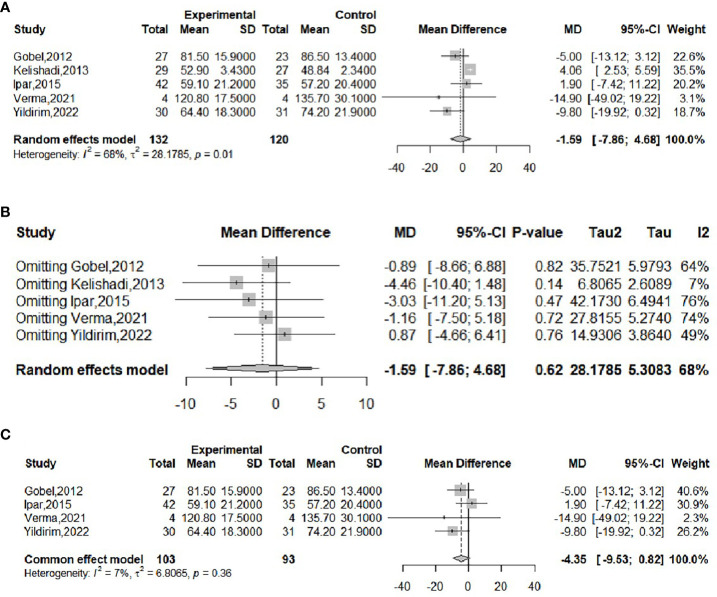
Forest plot of the effect of probiotic supplementation on weight **(A)**. Sensitivity analysis of the effect of probiotic supplementation on weight **(B)**. Forest plot of the effect of probiotic supplementation on weight (after elimination) **(C)**.

The effect of probiotics on weight was analyzed by five studies (16, 17, 20, 21, 23), 132 in the experimental group and 120 in the control group. The meta-merge heterogeneity was significant (*I^2^* = 68%, *P*=0.01), and the random effect model was applied to merge, as shown in **Figure 3A**. The WMD and 95% CI were 1.59 (-7.86, 4.68) kg, suggesting that the probiotic intervention did not have a significant effect on weight. To find the source of heterogeneity, sensitivity analysis was performed and found that heterogeneity was weakened by excluding the Kelishadi study (21) (**Figure 3B**). After excluding this study, the heterogeneity was *I^2^* = 7%, *P*=0.36, and the fixed effect model was applied to merge the two studies, as shown in **Figure 3C**. The WMD and 95% CI were -4.35 (-9.53, 0.82) kg, respectively, indicating that the probiotic intervention had no significant effect on improving the weight of the obese adolescent. The results were consistent with the results before the exclusion of the Kelishadi study, suggesting that although heterogeneity was high, study sensitivity was low, and the results were more robust and trustworthy. Begg’s test (*P* = 0.3272) and Egger’s test (*P* = 0.064) suggested that there was no significant publication bias.

**Figure 4:**
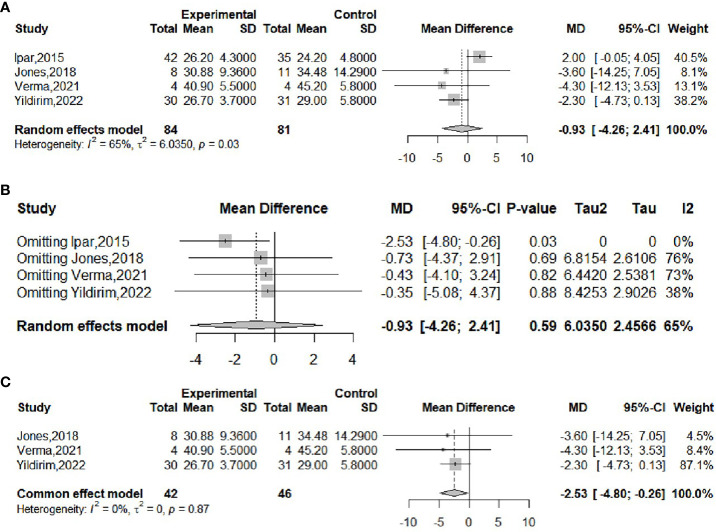
Forest plot of the effect of probiotic supplementation on BMI **(A)**. Sensitivity analysis of the effect of probiotic supplementation on BMI **(B)**. Forest plot of the effect of probiotic supplementation on BMI (after elimination) **(C)**.

The effect of probiotics on BMI was analyzed by four studies (16, 18, 20, 21), 84 in the experimental group and 81 in the control group. The meta-merge heterogeneity was significant (*I^2^* = 65%, *P*=0.03), and the random effect model was applied to merge, as shown in **Figure 4A**. The WMD and 95% CI were -0.93 (-4.26,2.41) kg/m^2^, suggesting that probiotic intervention had no significant effect on BMI. To find the source of heterogeneity, sensitivity analysis was performed and found that the heterogeneity was weakened by excluding the Ipar study (20) (**Figure 4B**). After excluding this literature, the heterogeneity was *I^2^* = 0%, *P*=0.87, which was combined by applying a fixed-effects model, as shown in **Figure 4C**, and the WMD and 95% CI were -2.53 (-4.8, -0.26) kg/m^2^, respectively, indicating that probiotic intervention was beneficial for the BMI of obese adolescents. This result is completely different from the results before the exclusion of the Ipar study, suggesting that the literature is highly sensitive and the results less robust. Begg’s test (*P* = 1) and Egger’s test (*P* = 0.4802) suggested that there was no significant publication bias.

**Figure 5:**
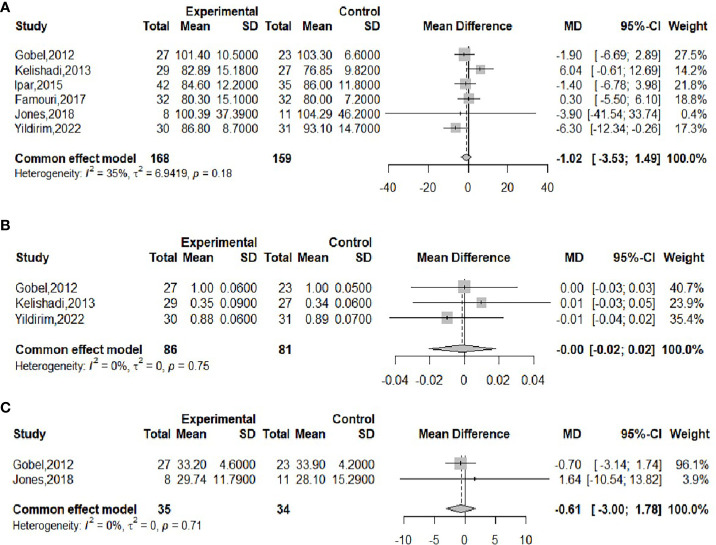
Forest plot of the effect of probiotic supplementation on WC **(A)**, WHR **(B)**, body fat% **(C)**.

The effect of probiotics on WC was analyzed by six studies (16, 18–21, 23), including 168 cases in the experimental group and 159 cases in the control group. There was no significant heterogeneity after performing meta-merge (*I^2^* = 35%, *P*=0.18), and the fixed effect model was applied for the merge, as shown in **Figure 5A**. The WMD and 95% CI were -1.02 (-3.53, 1.49) cm, respectively, which indicated that the probiotic intervention did not have a significant effect on WC in obese adolescents. Begg’s test (*P* = 0.3476) and Egger’s test (*P* = 0.9032) indicated no significant publication bias.

The effect of probiotics on WHR was analyzed by three studies (16, 21, 23), 86 in the experimental group and 81 in the control group. No significant heterogeneity was observed after performing meta-merge (*I^2^* = 0%, *P* = 0.75), and the fixed-effects model was applied for merging, as shown in **Figure 5B**, with WMD and 95% CI 0.00 (-0.02,0.02), indicating that there was no significant effect of probiotic interventions on the WHR of the obese adolescents. Begg’s test (*P* = 0.6015) and Egger’s test (*P* = 0.5549) indicated no significant publication bias.

The effect of probiotics on body fat% was analyzed by two studies (18, 23), 35 in the experimental group and 34 in the control group. There was no significant heterogeneity after performing meta-merge (*I^2^* = 0%, *P*=0.71), and the fixed effect model was applied for merging, as shown in **Figure 5C**, with WMD and 95% CI -0.61 (-3.00, 1.78)%, which indicated that probiotic interventions did not have a significant effect on the body fat % of the obese adolescents. Due to the small number of studies, no publication bias test was performed.

#### 3.4.2 The effect of probiotics on inflammatory markers (CRP, IL-6, TNF-α, and FC)

**Figure 6:**
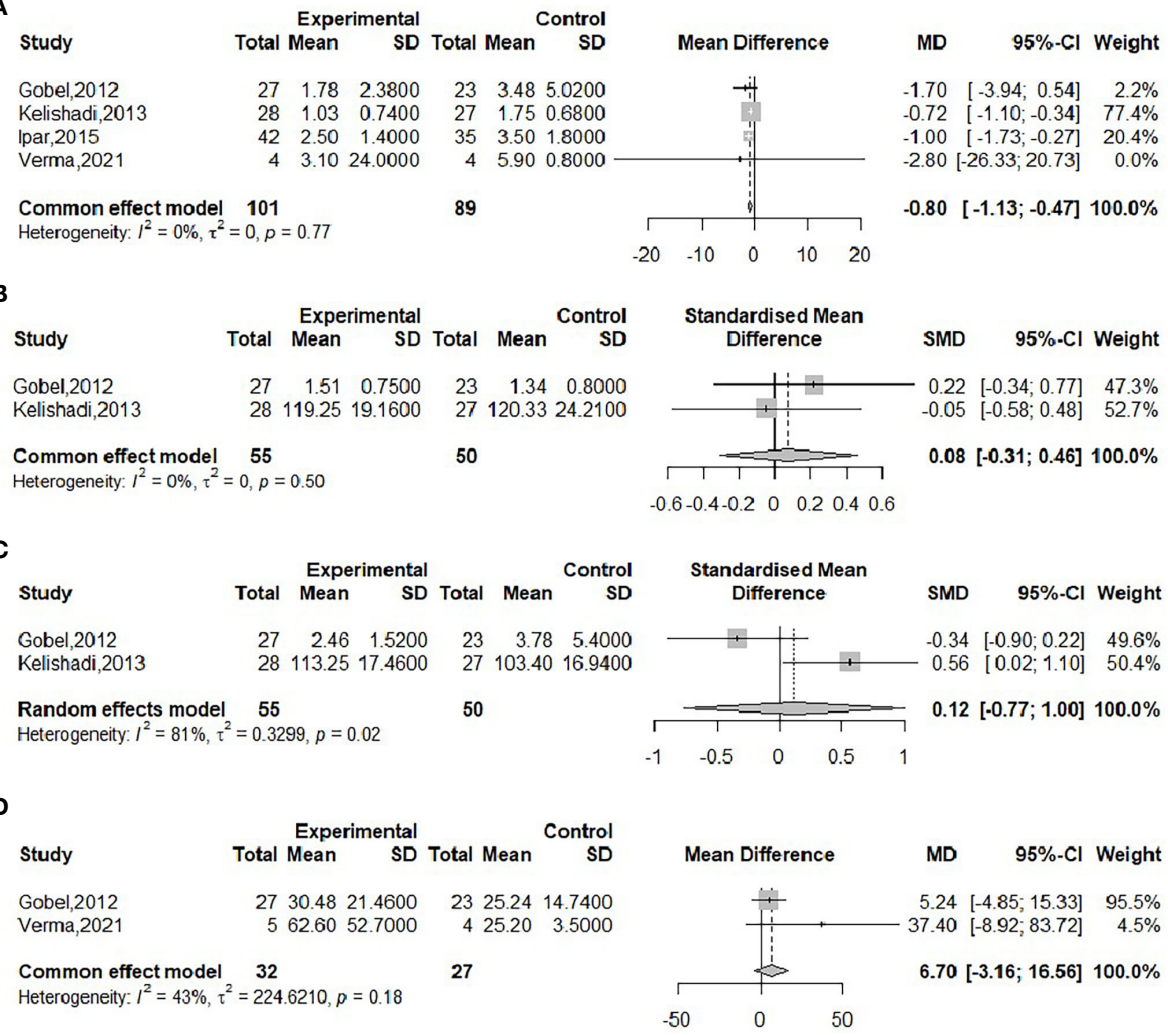
Forest plot of the effect of probiotic supplementation on CRP **(A)**, IL-6 **(B)**, TNF-α **(C)**, FC **(D)**.

The effect of probiotics on CRP was analyzed by four studies (17, 20, 21, 23), 101 in the experimental group and 89 in the control group. There was no significant heterogeneity after performing meta-merge (*I^2^* = 0%, *P* = 0.77), and the fixed-effects model was applied for merging, as shown in **Figure 6A**, with WMD and 95% CI -0.80 (-1.13, -0.47) mg/L, which indicated that probiotic interventions could reduce CRP levels in obese adolescents. Begg’s test (*P* = 0.4969) and Egger’s test (*P* = 0.2276) indicated no significant publication bias.

The effect of probiotics on IL-6 in obese adolescents was analyzed by two studies (21, 23), 55 in the experimental group and 50 in the control group. There was no significant heterogeneity after performing meta-merge (*I^2^* = 0%, *P*=0.50), applying the fixed effect model for merging, and because of its inconsistent units, effect sizes were merged with SMD, as shown in **Figure 6B**, with SMD and 95% CI 0.08 (-0.31, 0.46), respectively, indicating that probiotic intervention had no effect on IL-6. Due to the small number of included studies, a publication bias test was not performed.

The effect of probiotics on TNF-α in obese adolescents was analyzed by two studies (21, 23), 55 in the experimental group and 50 in the control group. The meta-merger heterogeneity was significant (*I^2^* = 81%, *P*=0.02). Due to the small number of included studies, sensitivity analysis and subgroup analysis were not performed, so the random effect model was applied to merge, and because of its inconsistent units, the effect size was merged with SMD, as shown in **Figure 6C**. The SMD and the 95% CI were 0.12 (-0.77, 1.00), respectively, indicating that probiotic intervention had no effect on TNF-α. Due to the small number of included studies, a publication bias test was not performed.

The effect of probiotics on FC in obese adolescents was analyzed by two studies (17, 23), 32 in the experimental group and 27 in the control group. No significant heterogeneity was observed after performing meta-merging (*I^2^* = 43%, *P*=0.18), and the fixed effect model was applied for merging, as shown in **Figure 6D**, with a WMD and 95% CI of 6.70 (-3.16, 16.56) mg/kg, which indicated that probiotic intervention had no effect on FC levels. The small number of included studies was not tested for publication bias.

#### 3.4.3 The effect of probiotics on FBG, RI, and HOMA-IR

**Figure 7:**
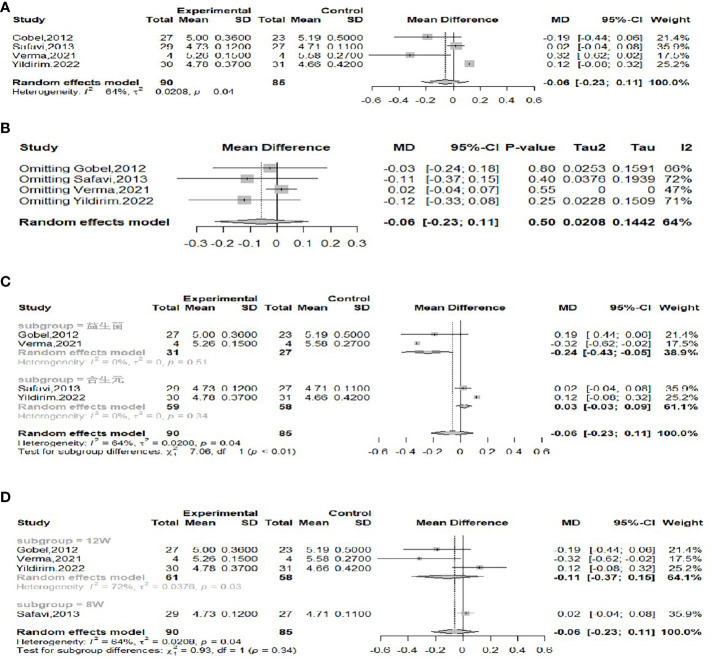
Forest plot of the effect of probiotic supplementation on FBG **(A)**. Sensitivity analysis of the effect of probiotic supplementation on FBG **(B)**. Subgroup analysis based on the type of intervention (probiotic/synbiotic) **(C)**. Subgroup analysis based on duration of intervention (8weeks/12weeks) **(D)**.

The effect of probiotics on FBG in obese adolescents was analyzed by four studies (16, 17, 22, 23), 90 in the experimental group and 85 in the control group. Significant heterogeneity (*I^2^* = 64%, *P*=0.04) was observed when meta-merging was performed (**Figure 7A**), and a sensitivity analysis did not reveal a significant reduction in heterogeneity after excluding a particular piece of literature (**Figure 7B**), suggesting that the results of the included studies were relatively robust. To further look for sources of interstudy heterogeneity, subgroup analyses based on the type of intervention (probiotic or synbiotic) and duration of intervention (8 weeks/12 weeks) were performed. When grouped by intervention type (**Figure 7C**), two of the studies with intervention type of probiotic (17, 23) and two of the studies with intervention type of synbiotic (16, 22) were meta-merged separately, and there was no significant heterogeneity in either (*I^2^* = 0), suggesting that the intervention type might be the source of heterogeneity, with WMD and 95% CI in the probiotic group of -0.24 (-0.43, 0.05) mmol/L and WMD and 95% CI in the synbiotic group of 0.03 (-0.03, 0.09) mmol/L, respectively. This suggests that probiotic treatment can reduce FBG levels, while synbiotic treatment has no effect on FBG. When grouped by intervention duration (**Figure 7D**), three studies (16, 17, 23) had an intervention duration of 12 weeks, one study (22) had an intervention duration of 8 weeks, and there was no significant attenuation of heterogeneity after the merger, suggesting that interstudy heterogeneity was not related to intervention duration. Begg’s test (*P* = 0.1742) and Egger’s test (*P* = 0.3993) indicated no significant publication bias.

**Figure 8:**
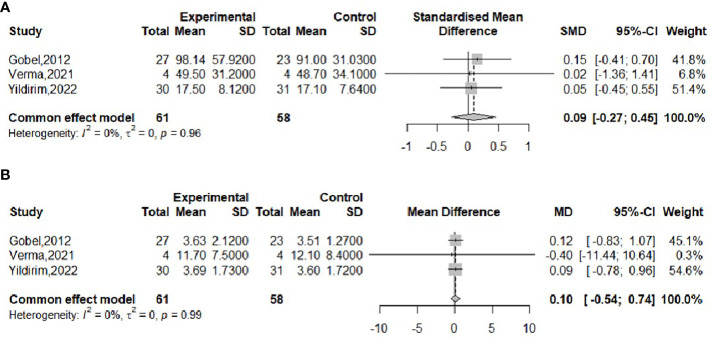
Forest plot of the effect of probiotic supplementation on RI **(A)**, HOMA-IR **(B)**.

The effect of probiotics on the RI in obese adolescents was analyzed in three studies (16, 17, 23), with 61 in the experimental group and 58 in the control group. No significant heterogeneity was observed when meta-merging was performed (*I^2^* = 0%, *P* = 0.96), the fixed-effects model was applied for merging, and the effect sizes were merged with SMD due to their inconsistent units, as shown in **Figure 8A**, with SMD and 95% CI 0.09 (-0.27,0.45), respectively, indicating that there was no effect of probiotic interventions on insulin levels. Begg’s test (*P* = 0.6015) and Egger’s test (*P* = 0.5112) indicated no significant publication bias.

The effect of probiotics on HOMA-IR in obese adolescents was analyzed by three studies (16, 17, 23), 61 in the experimental group and 58 in the control group. No significant heterogeneity was observed when meta-merging was performed (*I^2^* = 0%, *P*=0.99), and a fixed-effects model was applied for merging, as shown in **Figure 8B**, with a WMD and 95% CI of 0.10 (-0.54, 0.74), respectively, indicating that the probiotic intervention had no effect on HOMA-IR. Begg’s test (*P* = 0.6015) and Egger’s test (*P* = 0.3480) indicated no significant publication bias.

#### 3.4.4 The effect of probiotics on the lipid profile (TC, TG, LDL, HDL)

**Figure 9:**
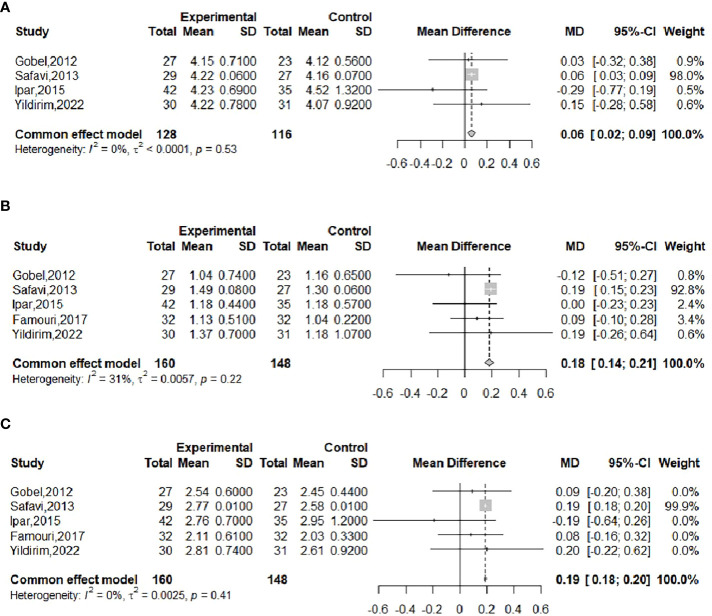
Forest plot of the effect of probiotic supplementation on TC **(A)**. Forest plot of the effect of probiotic supplementation on TG **(B)**. Forest plot of the effect of probiotic supplementation on LDL **(C)**.

The effect of probiotics on TC in obese adolescents was analyzed by five studies (16, 19, 20, 22, 23), of which the Famouri study (19) had a significantly higher TC in the intervention group than in the control group at baseline. This group was excluded, and the remaining four studies were combined (16, 20, 22, 23), with 128 cases in the experimental group and 116 cases in the control group. No significant heterogeneity was observed after performing meta-merging (*I^2^* = 0%, *P*=0.53), and the fixed-effects model was applied for merging, as shown in **Figure 9A**, with WMD and 95% CI 0.06 (0.02, 0.09) mmol/L, which indicated that probiotic interventions could elevate TC levels in adolescent obese adolescents. Begg’s test (*P* =0.1742) and Egger’s test (*P* =0.5417) indicated no significant publication bias.

The effect of probiotics on TG in obese adolescents was analyzed in five studies (16, 19, 20, 22, 23), with 160 in the experimental group and 148 in the control group. There was no significant heterogeneity after performing meta-merging (*I^2^* = 31%, *P* = 0.22), and the fixed-effects model was applied for merging, as shown in **Figure 9B**, with WMD and 95% CI: 0.18 (0.14, 0.21) mmol/L, which indicated that probiotic interventions could elevate TG levels in adolescent obese adolescents. Begg’s test (*P* = 0.6242) and Egger’s test (*P* = 0.0834) indicated no significant publication bias.

The effect of probiotics on LDL in obese adolescents was analyzed by five studies (16, 19, 20, 22, 23), including 160 cases in the experimental group and 148 cases in the control group, and there was no significant heterogeneity after meta-merging (*I^2^* = 0%, *P*=0.41). The fixed-effects model was applied to merge them, as shown in **Figure 9C**, and the WMD and 95% CI were 0.19 (0.18, 0.20) mmol/L, indicating that probiotic intervention can elevate LDL levels in adolescent obese patients. Begg’s test (*P* = 0.3272) and Egger’s test (*P* = 0.1077) indicated no significant publication bias.

**Figure 10:**
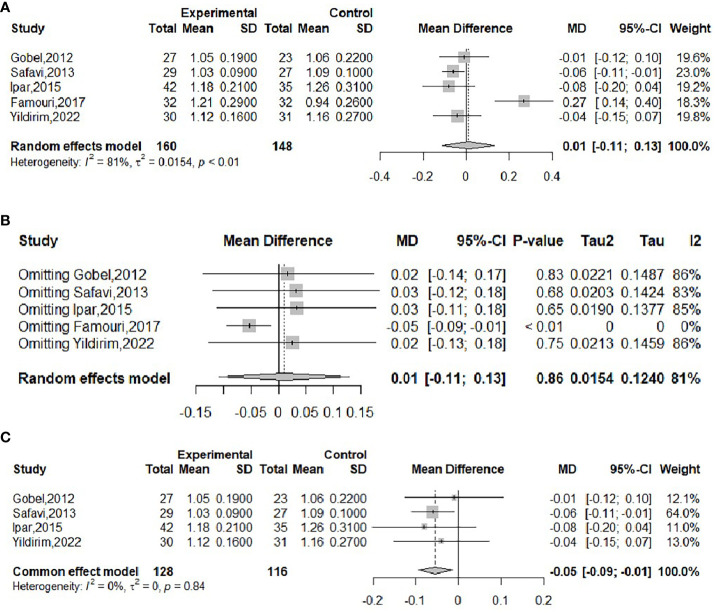
Forest plot of the effect of probiotic supplementation on HDL **(A)**. Sensitivity analysis of the effect of probiotic supplementation on HDL **(B)**. Forest plot of the effect of probiotic supplementation on HDL (after elimination) **(C)**.

The effect of probiotics on HDL in obese adolescents was analyzed in five studies (16, 19, 20, 22, 23), with 160 in the experimental group and 148 in the control group. Significant heterogeneity of meta-merge was observed (*I^2^* = 81%, *P*<0.01), and the random effects model was applied for the merge, as shown in **Figure 10A**, with WMD and 95% CI -0.01 (-0.11,0.13) mmol/L, suggesting that the probiotic intervention did not have a significant effect on HDL in obese adolescents. To find the source of heterogeneity, sensitivity analysis was performed and found that the heterogeneity was weakened after excluding the Famouri study (19) (**Figure 10B**). After excluding this literature, the heterogeneity was weakened (*I^2^* = 0%, *P*=0.84), and the fixed-effects model was applied for the merger, as shown in **Figure 10C**. The WMD and the 95% CI were -0.05 (-0.09, -0.01) mmol/L, respectively, indicating that the probiotic intervention could reduce HDL levels. This result is very different from the result before excluding the Famouri study, indicating high sensitivity and low robustness of the result. Begg’s test (*P* = 0.1416) and Egger’s test (*P* = 0.3392) indicated no significant publication bias.

## 4 Discussion

The WHO defines probiotics as nondigestible food ingredients that improve the health of the host by selectively stimulating the growth and/or activity of one or a limited number of bacterial species already established in the colon, with beneficial effects on the host (24). They are involved in host regulation in different ways: antagonizing the growth of pathogenic microorganisms and competitive adhesion to the intestinal mucosa and epithelium (antimicrobial activity), increasing intestinal mucus production and decreasing intestinal permeability (barrier function), and modulating the immune system of the gastrointestinal tract (immunomodulation) (25–27). All of these mechanisms influence the development of the microbiota to ensure the proper balance between the pathogen and the microorganisms needed for optimal host function. Furthermore, through a review of the literature we found that the most commonly researched and recommended probiotics include Lactobacillus genus and Bifidobacterium genus etc.

Our study found that probiotic intervention reduced BMI in overweight/obese adolescent patients but had no effect on metabolic indices such as weight, WC, WHR, or body fat%. Among them, no significant heterogeneity was found after meta-merging for WC, WHR, and body fat%, and greater heterogeneity was found for weight and BMI. To find the source of heterogeneity, sensitivity analyses were carried out, and all of them found that the heterogeneity was significantly reduced by exclusion of a particular piece of literature. Among them, the results of the effect on weight were the same before and after the exclusion, but the results of merging the results of BMI appeared to be inconsistent, which indicated that Ipar’s study (20) had a high sensitivity and that the results had a low robustness. Further analysis of Ipar’s study did not find clinical heterogeneity due to the same inclusion or exclusion criteria. When exploring methodological heterogeneity, we found that Ipar’s study had an open label design with a higher risk of bias compared to the rest of the included literature, so this group of data was excluded before combining them, and the combined results showed that probiotic interventions can reduce BMI levels in obese adolescent patients. The above findings are consistent with the results of a previous meta-analysis of children and adolescents (28), which did not analyze BMI together. In a multigroup meta-analysis of adults (29, 30), a significant effect of the application of probiotic ingredients in reducing body weight and BMI was observed. Adolescents are in a special growth and development stage, and during probiotic intervention tracking, adolescents are often accompanied by a simultaneous increase in weight and height, so we did not directly observe a clear decrease in body weight through probiotic intervention, but the decrease in BMI also indirectly reflected the benign effect of probiotics on body weight, and meaningful meta-results were obtained.

In this study, we found that probiotic intervention significantly reduced serum levels of CRP in overweight/obese adolescent patients, while it had no significant effect on IL-6, TNF-α, or FC. CRP, IL-6, TNF-α, and FC are indicators used to reflect the level of inflammation. The low-grade inflammatory response is a key factor in the development of obesity, and many studies have demonstrated that obese patients have an increase in proinflammatory cytokines (31). Previous studies on the effects of probiotic supplementation on inflammatory markers in adult obese patients have also found significant reductions in CRP (32). To our knowledge, this is the first meta-analysis to specifically assess the effect of probiotic supplementation on inflammatory markers in obese adolescents and observe a significant reduction in CRP, and more multicenter, larger sample size clinical trials are needed to demonstrate the efficacy of probiotic supplementation on inflammatory markers in obese adolescents, which in turn can help better guide clinical practice.

Our study found that probiotic intervention had no effect on improving insulin levels or insulin resistance in overweight/obese adolescent patients. Due to significant heterogeneity in the results of the effect on fasting blood glucose, subgroup analyses were performed to show that the heterogeneity was attenuated by grouping based on the type of intervention and that a reduction in fasting blood glucose was observed in the group supplemented with probiotics alone. In some previous studies, the same benefits of probiotic supplementation on glycemic control in patients were found (33–35). The mechanism may be related to the regulation of glucose metabolism by intestinal flora and its products, for example: 1. Probiotics can increase the secretion of glucagon-like peptide 1 (GLP-1) in the body. GLP-1 is an endogenous gut hormone secreted by L-cells that is key to promoting insulin secretion through entero-proteinotropic effects (36). Several studies have demonstrated the efficacy of GLP-1 agonists (liraglutide) for weight loss in pediatric patients (37). Specifically, probiotics promote GLP-1 secretion through three pathways. First, probiotics are able to produce short-chain fatty acids (SCFAs) by fermenting dietary fiber in the diet, which can promote GLP-1 production (38). Second, probiotics can also indirectly stimulate GLP-1 secretion by fermenting indigestible polysaccharides (39). Third, probiotics convert primary bile acids into secondary bile acids, which activate Takeda G protein receptor 5 and subsequently stimulate GLP-1 secretion (40). 2. Probiotics can improve mitochondrial damage. In animal experiments, lactobacilli were found to improve the morphological structure of mitochondrial damage caused by hyperglycemia. Improved mitochondrial health restored fatty acid β-oxidation, thereby reducing fatty acid accumulation in the liver and improving systemic glucose metabolism (41).

In this study, we found that probiotic intervention adversely affected TC, TG, and LDL in overweight/obese adolescent patients. Because of the significant heterogeneity in the combination of HDL, we further performed sensitivity analyses, and although the results showed that the exclusion of Famouri’s study reduced the heterogeneity, the study did not have a high risk of bias and did not find clinical, methodological or statistical heterogeneity, so we concluded that we could not exclude the data from this group of studies. The final results showed no effect of probiotic intervention on HDL in overweight/obese adolescent patients. Dyslipidemia is an important risk factor for obesity, which may involve elevated levels of TC, LDL, and TG and decreased levels of HDL (42). Past studies have shown that probiotics can improve lipid disorders, such as lowering blood cholesterol levels and improving the antioxidant capacity of LDL (43). Several mechanisms have been proposed for probiotics to lower cholesterol by controlling cholesterol metabolism: 1. Enzymatic action through bile salt hydrolase (BSH) of probiotics. BSH is an enzyme that catalyzes the hydrolysis of glycine and/or taurine-coupled bile salts to amino acid residues and free bile acids (44). Since coupled bile salts are more hydrophilic and are readily absorbed by the gastrointestinal tract, free bile acids are less soluble than coupled bile salts, are less likely to be reabsorbed by the intestinal tract and are more likely to be excreted in the feces (44), which will increase the need to synthesize new bile acids to replace those lost. Since cholesterol is a precursor for the resynthesis of new bile acids, the use of cholesterol to synthesize new bile leads to a decrease in blood cholesterol concentration. 2. The anabolic effect of probiotics on cholesterol in the small intestine can lower serum cholesterol by reducing the absorption of cholesterol in the intestine (45). Some probiotics can produce exopolysaccharides that adhere to cell surfaces and can absorb cholesterol (45). Very little of the cholesterol that is absorbed by the bacterial cells during growth in the small intestine is absorbed by the enterohepatic circulation (43) and thus may lead to lower serum cholesterol in humans. 3. Probiotics can also incorporate cholesterol into cell membranes, lowering blood cholesterol levels (46). 4. Probiotics convert cholesterol to fecal steroids through cholesterol reductase, thus reducing cholesterol absorption and eliminating it from the body with feces (47). 5. Probiotics inhibit cholesterol resynthesis by producing short-chain fatty acids (48). A previously conducted meta-analysis of the effects of a probiotic intervention on lipid profiles found significant reductions in TC, TG and LDL (49). In addition, there are still several studies (50–52) showing that probiotic supplementation in obese or overweight populations significantly reduces TC and LDL, but there is no significant difference in changes in HDL and TG. All these studies have confirmed the beneficial effects of probiotics in regulating the lipid profile, whereas our study found that probiotic intervention had an adverse effect on the regulation of lipids, which is contrary to previous conclusions. To determine the reason for this result, we further analyzed the trials included in the present study and found that the hypolipidemic effect of probiotics was found in all the studies, except for the studies of Gobel’s and Yildirim’s, which did not find any effect of probiotics on lipids. Therefore, we analyzed that the reason for this result may be because for the randomized controlled trials, we considered that the difference between the baseline level of the test group and the control group before the intervention was not statistically significant, so the data of the studies included in the meta-analysis were the data after the intervention of the two groups, whereas the results of the original studies usually compare the difference in the value of the change between the test group and the control group before and after the treatment, and due to the small number of related studies at the moment, for the results of the study, we still need to discuss the credibility of the results of the study further. With differences in experimental design, participant characteristics, and probiotic strains used between studies also contributing to differences in trial results, more well-designed RCTs are still needed to conclusively determine whether the use of probiotic strains is effective in improving lipid levels in obese adolescents.

Thus far, we searched for a meta-analysis (28) published in 2019 on the effectiveness of probiotic interventions on overweight/obesity in adolescents, which applied the means and standard deviations of the values of changes in anthropometric and metabolic indices at baseline and at the end of the intervention and did not find any good effect of the application of probiotic-based supplements in terms of the improvement of waist circumference, body weight, body fat percentage, fasting glucose, and lipid profile (LDL, HDL, TC, TG). After reviewing the relevant literature, we concluded that the included studies were randomized controlled trials, that there were no significant differences in the effect indicators at baseline, so postintervention means and standard deviations were applied, and that the meta-analysis described above was published in 2019, whereas we reviewed relevant RCTs up to 2023. Therefore, there were some differences in the included RCTs, and unlike the results of the above studies, in addition to the above effect indicators, we analyzed the effects of probiotics on parameters including BMI, WHR, insulin, and HOMA-IR, CRP, IL-6, TNF-α, and FC and found a beneficial effect of probiotic interventions in decreasing the effect indicators, such as BMI, FBG, and CRP, in adolescent obese patients. From this, we concluded that probiotic interventions may have a positive role in improving metabolic and inflammatory markers in adolescent obese patients. The main limitations of our meta-analysis included the diversity of probiotic interventions used in different studies, the subgroups of studies and the duration of participant follow-up, and due to the small number of studies, we were unable to group studies by probiotic strain and duration of intervention follow-up. In addition, the effect of confounding variables, including dietary patterns and lifestyle, intraindividual strain differences, and individual genotypes, on the effectiveness of probiotic interventions is unknown, and future studies may analyze this in more detail based on patient and intervention characteristics.

## 5 Conclusion

According to our results, probiotic supplementation was beneficial in managing metabolic indicators such as fasting blood glucose, body mass index and inflammation-related C-reactive protein in overweight or obese adolescents. Further large scale studies are warranted to confirm present findings and to identify the effects and mechanisms to provide more precise evidence for clinical intervention.

## Data availability statement

The original contributions presented in the study are included in the article/supplementary material. Further inquiries can be directed to the corresponding authors.

## Author contributions

YD: Writing – original draft. LN: Writing – original draft. XZ: Writing – review & editing. LW: Writing – review & editing. YM: Software, Writing – review & editing.
